# Nintedanib modulates type III collagen turnover in viable precision-cut lung slices from bleomycin-treated rats and patients with pulmonary fibrosis

**DOI:** 10.1186/s12931-022-02116-4

**Published:** 2022-08-04

**Authors:** Christina Hesse, Valerie Beneke, Sebastian Konzok, Claudia Diefenbach, Jannie Marie Bülow Sand, Sarah Rank Rønnow, Morten Asser Karsdal, Danny Jonigk, Katherina Sewald, Armin Braun, Diana Julie Leeming, Lutz Wollin

**Affiliations:** 1 Fraunhofer Institute for Toxicology and Experimental Medicine ITEM, Member of German Center for Lung Research (DZL), Hannover, Germany; 2 Biomedical Research in Endstage and Obstructive Lung Disease Hannover (BREATH), Member of Fraunhofer International Consortium for Anti-Infective Research (iCAIR), Member of Fraunhofer Cluster of Excellence Immune-Mediated Diseases (CIMD), Hannover, Germany; 3 Translational Medicine + Clinical Pharmacology, Boehringer Ingelheim Pharma GmbH & Co. KG, Biberach, Germany; 4 Nordic Bioscience A/S, Biomarkers & Research, Herlev, Denmark; 5 Institute of Pathology, Hannover Medical School, Hannover, Germany; 6 Biomedical Research in Endstage and Obstructive Lung Disease Hannover (BREATH), Member of German Center for Lung Research (DZL), Hannover, Germany

**Keywords:** Antifibrotic therapy, Collagen, Extracellular matrix, Human lung, Precision-cut lung slices, Neoepitope biomarkers, Nintedanib, Pirfenidone, Pulmonary fibrosis

## Abstract

**Background:**

Aberrant extracellular matrix (ECM) deposition and remodelling is important in the disease pathogenesis of pulmonary fibrosis (PF). We characterised neoepitope biomarkers released by ECM turnover in lung tissue from bleomycin-treated rats and patients with PF and analysed the effects of two antifibrotic drugs: nintedanib and pirfenidone.

**Methods:**

Precision-cut lung slices (PCLS) were prepared from bleomycin-treated rats or patients with PF. PCLS were incubated with nintedanib or pirfenidone for 48 h, and levels of neoepitope biomarkers of type I, III and VI collagen formation or degradation (PRO-C1, PRO-C3, PRO-C6 and C3M) as well as fibronectin (FBN-C) were assessed in the culture supernatants.

**Results:**

In rat PCLS, incubation with nintedanib led to a reduction in C3M, reflecting type III collagen degradation. In patient PCLS, incubation with nintedanib reduced the levels of PRO-C3 and C3M, thus showing effects on both formation and degradation of type III collagen. Incubation with pirfenidone had a marginal effect on PRO-C3. There were no other notable effects of either nintedanib or pirfenidone on the other neoepitope biomarkers studied.

**Conclusions:**

This study demonstrated that nintedanib modulates neoepitope biomarkers of type III collagen turnover and indicated that C3M is a promising translational neoepitope biomarker of PF in terms of therapy assessment.

**Supplementary Information:**

The online version contains supplementary material available at 10.1186/s12931-022-02116-4.

## Background

Pulmonary fibrosis (PF) encompasses a wide spectrum of lung diseases, characterised by scarring of the lung tissue and respiratory failure, and is generally associated with high mortality [1, 2]. The underlying mechanisms of PF are incompletely understood, but it is hypothesised that lung injury by external factors, such as smoking, environmental dusts or infections, triggers a dysregulated wound healing response; this can lead to uncontrolled deposition of extracellular matrix (ECM) proteins such as collagens, which causes destruction of the lung architecture [1–3]. Idiopathic pulmonary fibrosis (IPF) is the most common and severe idiopathic interstitial pneumonia (IIP) and has the worst prognosis among IIPs [4, 5].

On the molecular and cellular level, IPF is characterised by an excessive deposition of abnormal ECM and destruction of the original alveolar architecture, eventually leading to respiratory failure and death of the patient [3]. This remodelling of the ECM in IPF is accompanied by abnormalities in collagen turnover, including both fibrogenesis and fibrolysis, and is a hallmark of disease development and progression [6, 7]. As an example, the PROFILE study has shown that both elevated and rising serologically assessed levels of specific ECM neoepitope biomarkers were associated with disease progression and mortality in patients with IPF [6, 7].

To date, nintedanib and pirfenidone are the only approved antifibrotic treatments for IPF [8, 9]. Nintedanib is also approved for the treatment of chronic fibrosing interstitial lung diseases with a progressive phenotype and systemic sclerosis-associated interstitial lung disease [8]. Nintedanib is a small molecule tyrosine kinase inhibitor that targets fibroblast growth factor receptor 1–3, platelet-derived growth factor receptor α and β, vascular endothelial growth factor receptor 1–3, and multiple non-receptor tyrosine kinases, including the proto-oncogene tyrosine-protein kinase Src, tyrosine-protein kinase Lyn, lymphocyte-specific protein tyrosine kinase, Fms-like tyrosine kinase-3, colony-stimulating factor-1 receptor and several other tyrosine kinases [10, 11]. It has been shown to inhibit fibroblast proliferation, migration and transition to active myofibroblasts, as well as secretion of ECM [8]. Pirfenidone’s precise mode of action is unknown, but it is metabolised through the CYP1A2 enzyme pathway [9]. It regulates important fibrotic cytokines and growth factors and inhibits inflammatory mediators [12].

Disease management of IPF and PF in clinical practice remains challenging. There is a need for biomarkers to predict disease progression, inform treatment decisions and assess responses to antifibrotic therapy for IPF in clinical trials and clinical practice [6, 13]. One potential group of biomarkers that is being investigated in IPF are markers of ECM turnover [6, 7, 13]. The formation and degradation of ECM proteins create distinct newly formed epitopes known as neoepitopes that enter the circulation and thus are detectable as biomarkers of tissue turnover in the blood [6, 14, 15]. The neoepitope biomarkers included in the current study were the interstitial collagen synthesis markers PRO-C1, PRO-C3 and PRO-C6 for types I, III and VI collagen, respectively [16–18]. In addition, the interstitial collagen degradation marker C3M for type III collagen [19], and a biomarker of fibronectin remodelling FBN-C [20], were investigated.

The bleomycin model of PF is a well-characterised animal model that is widely used to investigate pulmonary fibrogenesis [1, 21]. Bleomycin is an anti-neoplastic drug which increases reactive oxygen species, leading to apoptosis of alveolar epithelial cells and PF [21, 22]. The bleomycin model has histological characteristics similar to human disease; however, there are differences in chronicity and pathogenesis, which means the bleomycin model does not mimic all features of human PF [1, 21, 22].

We wanted to characterise potential neoepitope biomarkers in living models of PF and hypothesised that antifibrotic drugs may affect their turnover. The precision-cut lung slice (PCLS) technique allows fibrosis and the effect of antifibrotic drugs to be studied in a clinically relevant in vivo-like model that preserves microanatomy, local immunology, and cell–cell and cell–ECM interactions [1, 15]. The inclusion of human PCLS in this study provided the unique opportunity to work directly with parenchymal lung tissues from donors with end-stage IPF and PF, which are otherwise difficult to study, and to perform preclinical experiments with antifibrotic drugs in a highly translational model system.

The aim of this study was to link ECM neoepitope biomarkers found in lung tissue from animal models to patient lung tissue and to clinical trial data from other studies. We characterised neoepitope biomarkers in supernatants from the PCLS of bleomycin-treated rats and peripheral lung tissue from patient donors with PF and investigated the effect nintedanib and pirfenidone had on their accumulation.

## Materials and methods

### Preparation of PCLS from bleomycin-treated rats

Nineteen 6-month-old female Sprague–Dawley rats were housed at the animal facilities at Nordic Bioscience A/S, Denmark. Twelve rats were induced twice with bleomycin (0.25 mL/kg) by intratracheal installation using a MicroSprayer® Aerosolizer 2 days apart to cause PF. As controls, seven rats were induced with saline. Lungs were excised 14 days after the last dose of bleomycin/saline. Rat lung tissue was prepared as described previously [15]. Prior to euthanasia, the rats were anaesthetised and a small catheter was inserted into the trachea to fill the lungs with a 1.5% agarose solution to keep them dilated. Cores with an approximate diameter of 8 mm were punched from the lung tissues and PCLS of ~ 300 µm thickness were prepared using the Krumdieck tissue slicer MD4000 (TSE Systems, Berlin, Germany) in the presence of ice-cold Krebs–Henseleit buffer containing 25 mM glucose, 10 mM HEPES and 25 mM NaHCO_3_. The PCLS were transferred to wells for a wash-out period of minimum 1 h. Subsequently, PCLS were transferred to new wells and incubated with one slice per well in 48-well plates in 300 µL William’s Medium E with Glutamax (ThermoFisher Scientific, Waltham, MA, USA) containing 25 mM glucose and 50 µL/mL gentamicin (both Sigma-Aldrich, St Louis, MO, USA), with or without nintedanib (cat. no. 656247-17-5, Kemprotec Ltd., Smailthorn, UK) (0.01 µM, 0.03 µM, 0.1 µM and 0.3 µM) for 48 h. After the 48-h incubation period, supernatants were collected and stored at − 20 °C until the concentrations of PRO-C1 and C3M were measured. The Animal Ethics Committee of the Danish Ministry of Justice approved the experiment (2011/561-2003, 2021-15-2934-00467).

### Preparation of PCLS from patients with PF

Primary human lung tissue from the peripheral lung was provided by the Hannover Medical School (MHH, Hannover, Germany) from five male and five female patients with PF aged 39–75 years who underwent lung transplantation. The patient diagnoses were: usual interstitial pneumonia (UIP) (n = 2), UIP/IPF (n = 3), UIP/IPF with alveolar fibroelastosis (n = 1), UIP/IPF and combined emphysema (n = 1), UIP and nonspecific interstitial pneumonia (NSIP) with combined emphysema (n = 1), UIP and NSIP due to primary extrapulmonary disease (n = 1), and NSIP with myogenic metaplasia (n = 1).

Human PCLS were generated as previously described [23]. Briefly, lobes from the donors were filled with 2% agarose (Sigma Aldrich) in Dulbecco’s modified Eagle’s medium/nutrient mixture F-12 Ham with 15 mM HEPES and l-glutamine without phenol red (DMEM/F12) (Gibco, Thermo Fisher Scientific) via the bronchi. Subsequently, ~ 8 mm diameter cores were punched from 2 to 5 different fibrotic regions of the solidified tissue and cut into slices of 300–400 µm on a microtome (Krumdieck Tissue Slicer, Alabama Research and Development, Munford, AL, USA) in Earle’s Balanced Salts Solution buffered with sodium bicarbonate (0.2%) (both Sigma Aldrich).

PCLS were washed separately for 2 h in culture medium consisting of DMEM/F12 containing 100 units/mL penicillin and 100 µg/mL streptomycin (both Sigma Aldrich) without foetal calf serum. They were then transferred into 24-well plates. Two PCLS were incubated per well in culture medium, and supernatant was pooled from two wells to give one sample. For exposure to test items, PCLS were incubated for 48 h under normal cell culture conditions (37 °C, 5% CO_2_) with nintedanib (Boehringer Ingelheim, Ingelheim, Germany) (0.01 µM, 0.03 µM, 0.1 µM and 0.3 µM) or pirfenidone (TCI Deutschland GmbH, Eschborn, Germany) (100 µM) in 500 µL (donor 1 and 2) or 250 µL (donor 3 to 10) culture medium in duplicate. These concentrations are within the range that can be achieved using standard dosing of nintedanib or pirfenidone in patients with IPF [24]. PCLS for the control groups were incubated in culture medium only, and originated from the same IPF donor and region as the nintedanib/pirfenidone slices in each case, without mixing regions.

Tissue culture supernatants were collected after incubation and supplemented with a 0.2% protease inhibitor cocktail (Sigma Aldrich), then frozen to − 80 °C before concentrations of neoepitope biomarkers were determined. For viability assessments, lactate dehydrogenase (LDH) release was assayed in the supernatants as described previously [25] using the LDH Cytotoxicity Detection Kit according to the manufacturer’s instructions (Roche, Basel, Switzerland). Triton X-100 (1% in phosphate buffered saline)-treated PCLS from the same donor were investigated as reference control (set to 100%; see Additional file 1: Fig. S1). If required, multiple dilutions of the samples were applied to adjust for high optical density values. Absorbance was measured at 490 nm and 630 nm as reference wavelengths using a microplate reader (Microplate Reader Infinite® 200 Pro Tecan Group, Männedorf, Switzerland).

The experiments with human lung tissue were approved by the ethics committee of the Hannover Medical School and are in accordance with the Code of Ethics of the World Medical Association (renewed on 22.04.2015, number 2701-2015). All patients or their next of kin, caregivers, or guardians gave written informed consent for using lung tissue for research. All information regarding the identity of the patients was anonymised.

### Biomarker assessments

Levels of neoepitope biomarkers were determined in rat and/or patient tissue PCLS supernatants using specific competitive enzyme-linked immunosorbent assays as previously described for type I, type III and type VI collagen formation (PRO-C1 [16], PRO-C3 [17] and PRO-C6 [18]), type III collagen degradation (C3M) [19] and soluble fibronectin (FBN-C [20]). Each assay employed a monoclonal antibody specific for the neoepitope in question. In brief, 96-well streptavidin-coated plates were incubated with 100 µL biotinylated peptide for 30 min at 20 °C, with shaking at 300 rpm. After 5× wash in washing buffer (20 mM Tris, 50 mM NaCl, pH 7.2), 20 µL of calibrator peptide, quality control samples or PCLS supernatants were added to appropriate wells, followed by 100 µL horse radish peroxidase- labelled monoclonal antibody, and incubated with shaking at 300 rpm according to assay specification at 4 °C or 20 °C for 1–20 h. Following 5 × wash in wa shing buffer, 100 µL tetramethylbenzidine was added and plates were incubated for 15 min at 20 °C in the dark, with shaking at 300 rpm. To stop the reaction, 100 µL 1% H_2_SO_4_ was added, and the plates were read in an enzyme-linked immunosorbent assay reader at 450 nm, with 650 nm as a reference. A calibration curve was plotted using a 4-parametric mathematical fit model. Supernatants were kept frozen until use, and were assessed in single determinations for rat PCLS and double determinations for human PCLS. Coefficient of variability percentages on double determinations were approved if under 15%. Values below the measurement range were assigned the lower limit of detection of the assay (C3M 0.011 ng/mL; PRO-C1 2.3 ng/mL; PRO-C3 0.5 ng/mL; PRO-C6 0.15 ng/mL; FBN-C 7.61 ng/mL).

### Statistical analysis

It was assumed that there would be non-uniform, patchy distribution of fibrotic lesions in donor lungs and different grades of fibrosis severity in the sliced tissue cores from different regions of a donor lung. Thus, the absolute change in neoepitope biomarker concentration in the supernatant of incubated slices was normalised by subtracting the mean concentration of the control (medium only) slices of each corresponding tissue region from each donor. The half- maximal inhibition values (EC_50_) were calculated by plotting the percentage change of the neoepitope biomarker concentration against the log of the nintedanib concentration using an asymmetric (five parameter) least squares fit (GraphPad, 9.00; GraphPad Software, Inc., La Jolla, CA, USA).

For statistical analysis, absolute neoepitope biomarker concentrations were compared against medium control for PCLS from patients with PF and against bleomycin-treated rat PCLS for the animal experiments. Statistical differences between groups were analysed by Kruskal–Wallis test followed by Dunn’s multiple comparison test for nonparametric data (GraphPad Prism 9.00). P < 0.05 was considered statistically significant.

## Results

### Nintedanib reduces C3M in the supernatant of viable lung slices from bleomycin-treated rats

**Fig. 1:**
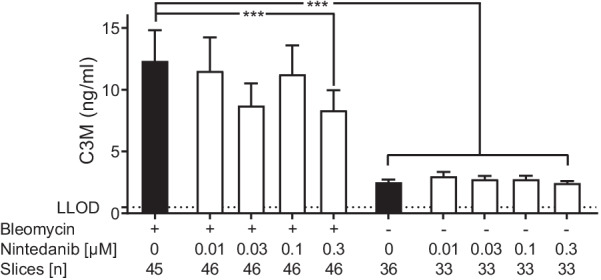
Nintedanib reduced C3M concentrations in the PCLS supernatant from bleomycin in vivo treated rats. C3M concentrations from PCLS from bleomycin-treated rats incubated with nintedanib for 48 h compared with PCLS from vehicle-treated rats. Number of slices indicates the total number from all rats evaluated at that dose. Statistical differences between groups were analysed by Kruskal–Wallis test followed by Dunn’s multiple comparison test for nonparametric data. Data are shown as mean ± SEM. ***P < 0.001. *C3M* neo-epitope of MMP-9 mediated degradation of type III collagen, *LLOD* lower limit of detection, *PCLS* precision-cut lung slices, *SEM* standard error of the mean

From the seven saline-treated rats, a total of 33 viable lung slices were prepared for each concentration of nintedanib and 36 for the vehicle. From the 12 bleomycin-treated rats, a total of 46 viable lung slices were prepared for each concentration of nintedanib and 45 for the vehicle. We showed that there was a significant fivefold increase in C3M in the supernatant of PCLS (12.42 ± 1.87 ng/mL) compared with vehicle control (2.47 ± 0.11 ng/mL) (P < 0.001) after the 48-h incubation period (Fig. 1). Incubation with 0.3 µM nintedanib led to a statistically significant maximum reduction of C3M by 40% (P < 0.001) (Fig. 1). Nintedanib had no effect on PRO-C1, the other neoepitope biomarker investigated (data not shown).

### Nintedanib reduces type III collagen neoepitope biomarkers in the supernatant of viable lung slices from patient donors with PF

All tested neoepitope biomarkers were detectable in control PCLS from patients with PF within range.

**Fig. 2:**
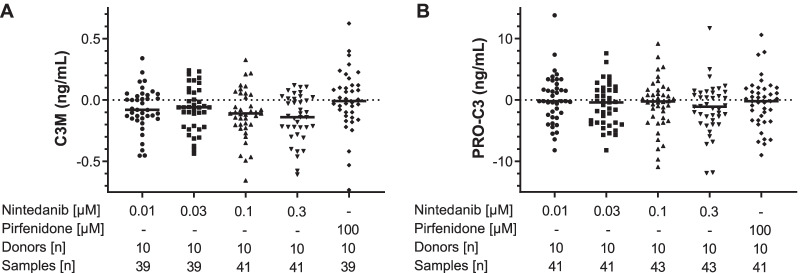
Nintedanib reduced C3M and PRO-C3 concentrations in the PCLS supernatant from patients with PF. **a** Absolute changes in C3M and **b** PRO-C3 concentrations from PCLS incubated with nintedanib and pirfenidone for 48 h compared with PCLS incubated with medium. Samples were generated by pooling supernatant from two wells, with each well containing two PCLS. Statistical differences between groups were analysed by Kruskal–Wallis test followed by Dunn’s multiple comparison test for nonparametric data. Two data points in part A and three in part B lie outside the plotted axes. *C3M* neo-epitope of MMP-9 mediated degradation of type III collagen, *PCLS* precision-cut lung slices, *PF* pulmonary fibrosis, *PRO-C3* ADAMTS-2 mediated release of the N-terminal pro-peptide of type III collagen

**Fig. 3:**
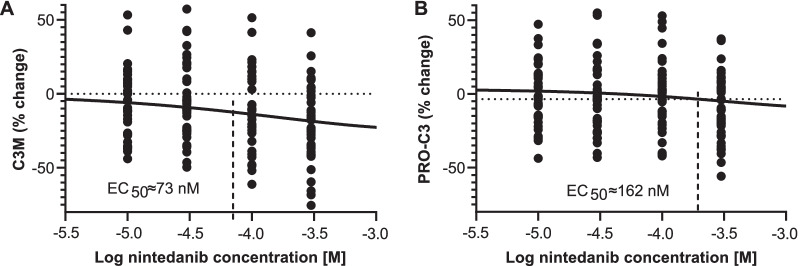
Inhibition of C3M and PRO-C3 in the PCLS supernatant from patients with PF. Percentage changes in **a** C3M and **b** PRO-C3 concentrations from PCLS incubated with nintedanib compared with PCLS incubated with medium. The EC_50_ were cal culated with asymmetric (five parameter) least squares fit. Six data points lie outside the plotted axes. C3M*C3M* neo-epitope of MMP-9 mediated degradation of type III collage n; *EC*_50_ half- maxi mal inhibition values; *PCLS* precision-cut lung slices; *PF* pulmonary fibrosis; *PRO-C3* ADAMTS-2 mediated release of the N-terminal pro-peptid e of type III collagen

Incubation of PCLS from patients with PF with nintedanib reduced C3M supernatant levels compared with supernatant from media-only controls in a concentration-dependent manner (Fig. 2a). This reached statistical significance (P < 0.05) at the highest concentration of nintedanib (0.3 µM), corresponding to a reduction in C3M concentration of 24%. The EC_50_ by nintedanib of C3M was ≈ 73 nM (Fig. 3a). In addition, incubation with nintedanib led to a small (9%) but concentration-dependent trend to reduced PRO-C3 compared with media-only controls (Fig. 2b) (EC_50_ ≈ 162 nM, Fig. 3b). Absolute values of neoepitope biomarker concentrations are shown in Table 1. Incubation with pirfenidone had no effect on C3M and only marginal effects on PRO-C3. There were no effects of either nintedanib or pirfenidone on the levels of PRO-C1, PRO-C6 and FBN-C (Table 1).

**Table 1:** Absolute concentrations of neoepitope biomarkers in PCLS supernatant from patients with PF

	C3M			PRO-C1			PRO-C3			PRO-C6			FBN-C		
	Mean (ng/mL)	± SEM	n	Mean (ng/mL)	± SEM	n	Mean (ng/mL)	± SEM	n	Mean (ng/mL)	± SEM	n	Mean (ng/mL)	± SEM	n
Control^a^	0.75	0.05	41	27.11	4.39	29	15.07	1.16	43	1.22	0.19	27	47.96	9.34	38
Nintedanib 0.01 µM	0.65	0.04	39	22.94	4.16	27	14.75	1.49	41	1.13	0.22	25	45.90	11.18	35
Nintedanib 0.03 µM	0.70	0.05	39	20.33	3.93	27	13.79	1.09	41	1.05	0.19	25	41.85	10.65	37
Nintedanib 0.1 µM	0.63	0.05	41	25.29	3.93	29	14.42	1.11	43	1.07	0.17	27	45.46	9.86	38
Nintedanib 0.3 µM	0.57*	0.04	41	22.46	3.99	29	13.77	1.17	43	1.19	0.19	27	44.52	9.72	38
Pirfenidone 100 µM	0.72	0.06	39	23.61	3.95	27	14.28	1.02	41	1.25	0.20	25	45.61	9.46	36

^a^DMEM/F12 media containing antibiotics. Data are shown as mean extracellular matrix turnover marker concentration of all available supernatants of all incubated slices from all donors per group ± SD. C3M, PRO-C3 and FBN-C concentration was explored in (n) samples of 10 donors, PRO-C1 and PRO-C6 in (n) samples of 7 donors. Sufficient supernatant was not available to test all markers in some cases, so numbers vary. *P < 0.05 versus control. C3M, neo-epitope of MMP-9 mediated degradation of type III collagen; DMEM, Dulbecco’s modified Eagle’s medium/nutrient mixture; F-12, F-12 Ham; FBN-C, C-terminal of fibronectin; PCLS, precision-cut lung slices; PRO-C1 proteinase mediated release of the internal epitope in the N-terminal pro-peptide of type I collagen; PRO-C3, ADAMTS-2 mediated release of the N-terminal pro-peptide of type III collagen; PRO-C6, C-terminal of released C5 domain of type VI collagen α3 chain (endotrophin); SD, standard deviation; SEM, standard error of the mean

## Discussion

In this study, we characterised a panel of neoepitope biomarkers quantifying ECM remodelling in PCLS prepared from bleomycin-treated rats, as well as from patient donors with PF, and investigated the effects of pirfenidone and nintedanib on these biomarkers. Our main findings were: (1) in PCLS from rat fibrotic lung tissue, incubation with nintedanib reduced type III collagen degradation, assessed by C3M levels. PRO-C3 was not measured in our animal model, so we were unable to ascertain if there were any effects on this neoepitope biomarker; (2) in PCLS from patient donors with PF, nintedanib led to reductions in type III collagen formation and degradation, assessed by PRO-C3 and C3M levels in a concentration-dependent manner, with EC_50_ values for C3M and PRO-C3 at or slightly above the expected clinical exposure after standard dosing of 150 mg nintedanib twice daily in patients with IPF [26–28]. In addition, clinical studies have shown that PRO-C3 and C3M levels are elevated in the serum of patients with IPF compared with unaffected individuals and are biomarkers of IPF severity and progression [6, 7, 29]. Collectively, these data indicate that nintedanib modulates neoepitope biomarkers of type III collagen turnover by affecting both type III collagen formation and degradation. These results also indicate that C3M, a biomarker of type III collagen degradation, appears to be the most promising neoepitope biomarker linking data from animal model and donor PCLS studies with data from clinical trials in patients.

Type III collagen is an important interstitial collagen, which is expressed in healthy and fibrotic lung tissues [14, 30]. In our study, the effects of nintedanib we observed on both PRO-C3 and C3M indicate that the drug may affect pathological remodelling in PF by shifting the balance of type III collagen turnover in two ways: (1) by exerting antifibrotic effects on fibroblasts, leading to decreased PRO-C3 secretion and reduced fibrogenesis, and (2) by modulating matrix metalloproteinase (MMP) secretion and activity, leading to reductions in excessive ECM destruction and C3M release. Nintedanib has been shown to exert antifibrotic effects on human lung fibroblasts and significantly increased the activity and levels of MMP-2 [31]. In pericytes, nintedanib significantly increased MMP-8, MMP-9 and MMP-13 secretion, and MMP-2 and MMP-9 activity [32]. It may be expected that incubating PCLS with nintedanib would increase MMP activity and therefore lead to increased levels of C3M. Interestingly, we observed the opposite effect with exposure to nintedanib leading to a decrease in C3M, indicating that, at least at the 48-h time point in our model, nintedanib is not exerting its affect primarily through MMP activation. An analysis over multiple time points would be required to determine the exact kinetics of PRO-C3/C3M ratio after treatment of PCLS with nintedanib.

In addition to PRO-C3 and C3M, other neoepitope biomarkers have been implicated in disease progression and mortality in PF in clinical studies. In the PROFILE study, the neoepitope biomarkers PRO-C3, PRO-C6, C1M, C3M, C6M and C-reactive protein degraded by MMPs 1 and 8 (CRPM) were higher in patients with progressive versus stable IPF, indicating an association with disease progression. Rising levels of C1M, C3M, C6M and CRPM were associated with an increased risk of overall mortality [6, 7].

The INMARK study investigated changes in biomarkers of ECM turnover as predictors of disease progression and evaluated the effect of nintedanib treatment [13]. Patients with IPF were randomised 1:2 to receive 150 mg nintedanib twice daily or placebo for 12 weeks, followed by an open-label period during which all patients received nintedanib for 40 weeks. The study did not meet its primary endpoint of rate of change in CRPM in the intention-to-treat population, and there were no significant differences in the rate of change of C1M or C3M between the nintedanib and placebo arms at Week 12. However, in patients treated with nintedanib there was a significant reduction in C3M from baseline at Weeks 16 and 20, and the reduction in the placebo/nintedanib arm at Week 24 was similar to that at Week 12 [33]. In addition, baseline levels of CRPM and C3M were associated with disease progression over 52 weeks [34]. Compared with placebo, treatment with nintedanib significantly reduced PRO-C6 at Weeks 4 and 12, and significantly increased PRO-C3 at Week 4 but not at Week 12 [33]. Analyses of the long-term effects of nintedanib treatment on ECM turnover biomarkers are ongoing and could provide insights into a possible treatment response on neoepitope biomarkers.

In contrast to the INMARK study [33], we did not observe any effects of nintedanib on the type VI collagen neoepitope biomarker PRO-C6. The differences between the results of this study and those in INMARK may be explained by nintedanib exerting different effects in in vivo and ex vivo systems compared with those in patients. Neoepitope biomarkers were evaluated in patient serum in the INMARK study, and therefore would reflect the systemic effects of nintedanib treatment on ECM remodelling. In our PCLS system, type III collagen could be the main collagen being produced through the pathways that are affected by nintedanib.

In this study, we only analysed the effects of nintedanib at 48 h compared with several weeks’ exposure in the clinical studies. Potentially different results could be obtained by varying the incubation period.

In agreement with our data, nintedanib reduced the secretion of PRO-C3 levels by transforming growth factor-beta 1 (TGF-β1)-induced fibroblasts in an in vitro Scar-in-a-Jar assay [35]. However, unlike in our study, there was also an effect on the neoepitope biomarkers PRO-C1, PRO-C6 and FBN-C in the in vitro model. The differences between this and the present ex vivo study could be explained by differences between the disease models and methodologies, making it difficult to draw accurate comparisons. For example, the Scar-in-a-Jar assay is an in vitro cell culture model using TGF-β1-stimulated fibroblasts with cells initially seeded on plastic, whereas the PCLS system is a more complex model that is derived from multicellular tissue [23, 35].

In our study, incubation of patient PCLS with pirfenidone had only a marginal effect on PRO-C3 and no obvious effects on the other neoepitope biomarkers. Other studies have shown that pirfenidone exerts antifibrotic effects in models of PF, but only at concentrations of 1–2.5 mM [35, 36]. In our study, we used a concentration of 100 µM, which is within the expected physiological range after standard dosing with pirfenidone [24]. Another possible reason for the lack of effect of pirfenidone on the neoepitope biomarkers could be related to its mode of action, which has not been fully characterised.

The strengths of this study are that we investigated neoepitope biomarkers in both an animal model of PF and patient donor tissue using the PCLS technique, which preserves the three-dimensional structure of the tissue and is an established translational model for preclinical investigations [1, 23]. Limitations of this study are the small number of PF samples from donors with different subtypes of IIP, and who are likely to be treated with different therapy regimens, and the considerable variability between human lung slices from different patients and regions of the lung. In addition, unlike tissue collected from rat lungs, collection of patient donor tissue was restricted to peripheral regions of the lung, which could result in different target cells and tissues between our PCLS models. A limitation of the PCLS model is that it is a static model that would not replicate all the features in vivo, such as cellular infiltration. There were only limited tissue slices available, which restricted the number of neoepitope biomarkers that could be investigated in this study, and this meant that the number of sections was low for some neoepitope biomarkers.

## Conclusion

Nintedanib appears to modulate PRO-C3 and C3M, suggesting it exerts antifibrotic effects via the type III collagen remodelling pathways. Our study indicates that C3M is the most promising biomarker of treatment response to nintedanib, linking animal model and patient lung tissue PCLS studies to clinical studies in PF [6, 7]. Further work is needed to validate these biomarkers in patients with PF.

## Supplementary Information

**Additional file 1: Figure S1.** No detectable increase in LDH in the culture supernatant of human PCLS after antifibrotic treatment.

## Data Availability

The data sets used and/or analysed during the current study are available from the corresponding author on reasonable request.

## Abbreviations

C1M: Neo-epitope of MMP-2, -9, -13 mediated degradation of type I collagen
C3M: Neo-epitope of MMP-9 mediated degradation of type III collagen
C6M: Neo-epitope of MMP-2 mediated degradation of type VI collagen
CRPM: Neo-epitope of MMP-mediated degradation c-reactive protein
DMEM: Dulbecco’s modified Eagle’s medium/nutrient mixture
ECM: Extracellular matrix
EC_50_: Half-maximal inhibition values
FBN-C: C-terminal of fibronectin
F12: F-12 Ham
IPF: Idiopathic pulmonary fibrosis
IIP: Idiopathic interstitial pneumonia
LDH: Lactate dehydrogenase
MMP: Matrix metalloproteinase
NSIP: Nonspecific interstitial pneumonia
PCLS: Precision-cut lung slice
PF: Pulmonary fibrosis
PRO-C1: Proteinase mediated release of the internal epitope in the N-terminal pro-peptide of type I collagen
PRO-C3: ADAMTS-2 mediated release of the N-terminal pro-peptide of type III collagen
PRO-C6: C-terminal of released C5 domain of type VI collagen α3 chain (endotrophin)
TGF-β1: Transforming growth factor-beta 1
UIP: Usual interstitial pneumonia

## References

[1] Westra IM, Pham BT, Groothuis GM, Olinga P (2013). Evaluation of fibrosis in precision-cut tissue slices. Xenobiotica.

[2] Wynn TA (2011). Integrating mechanisms of pulmonary fibrosis. J Exp Med.

[3] Richeldi L, Collard HR, Jones MG (2017). Idiopathic pulmonary fibrosis. Lancet.

[4] Wong AW, Ryerson CJ, Guler SA (2020). Progression of fibrosing interstitial lung disease. Respir Res.

[5] Ley B, Collard HR, King TE (2011). Clinical course and prediction of survival in idiopathic pulmonary fibrosis. Am J Respir Crit Care Med.

[6] Organ LA, Duggan AR, Oballa E, Taggart SC, Simpson JK, Kang'ombe AR, Braybrooke R, Molyneaux PL, North B, Karkera Y (2019). Biomarkers of collagen synthesis predict progression in the PROFILE idiopathic pulmonary fibrosis cohort. Respir Res.

[7] Jenkins RG, Simpson JK, Saini G, Bentley JH, Russell AM, Braybrooke R, Molyneaux PL, McKeever TM, Wells AU, Flynn A (2015). Longitudinal change in collagen degradation biomarkers in idiopathic pulmonary fibrosis: an analysis from the prospective, multicentre PROFILE study. Lancet Respir Med.

[8] OFEV® (nintedanib): summary of product characteristics. https://www.ema.europa.eu/en/documents/product-information/ofev-epar-product-information_en.pdf.

[9] Esbriet (pirfenidone): summary of product characteristics. https://www.ema.europa.eu/en/documents/product-information/esbriet-epar-product-information_en.pdf.

[10] Hilberg F, Roth GJ, Krssak M, Kautschitsch S, Sommergruber W, Tontsch-Grunt U, Garin-Chesa P, Bader G, Zoephel A, Quant J (2008). BIBF 1120: triple angiokinase inhibitor with sustained receptor blockade and good antitumor efficacy. Cancer Res.

[11] Hilberg F, Tontsch-Grunt U, Baum A, Le AT, Doebele RC, Lieb S, Gianni D, Voss T, Garin-Chesa P, Haslinger C, Kraut N (2018). Triple angiokinase inhibitor nintedanib directly inhibits tumor cell growth and induces tumor shrinkage via blocking oncogenic receptor tyrosine kinases. J Pharmacol Exp Ther.

[12] Macias-Barragan J, Sandoval-Rodriguez A, Navarro-Partida J, Armendariz-Borunda J (2010). The multifaceted role of pirfenidone and its novel targets. Fibrogenes Tissue Repair.

[13] Maher TM, Stowasser S, Nishioka Y, White ES, Cottin V, Noth I, Selman M, Rohr KB, Michael A, Ittrich C (2019). Biomarkers of extracellular matrix turnover in patients with idiopathic pulmonary fibrosis given nintedanib (INMARK study): a randomised, placebo-controlled study. Lancet Respir Med.

[14] Karsdal MA, Nielsen MJ, Sand JM, Henriksen K, Genovese F, Bay-Jensen AC, Smith V, Adamkewicz JI, Christiansen C, Leeming DJ (2013). Extracellular matrix remodeling: the common denominator in connective tissue diseases. Possibilities for evaluation and current understanding of the matrix as more than a passive architecture, but a key player in tissue failure. Assay Drug Dev Technol.

[15] Hansen NU, Karsdal MA, Brockbank S, Cruwys S, Ronnow S, Leeming DJ (2016). Tissue turnover of collagen type I, III and elastin is elevated in the PCLS model of IPF and can be restored back to vehicle levels using a phosphodiesterase inhibitor. Respir Res.

[16] Leeming DJ, Larsen DV, Zhang C, Hi Y, Veidal SS, Nielsen RH, Henriksen K, Zheng Q, Barkholt V, Riis BJ (2010). Enzyme-linked immunosorbent serum assays (ELISAs) for rat and human N-terminal pro-peptide of collagen type I (PINP)—assessment of corresponding epitopes. Clin Biochem.

[17] Nielsen MJ, Nedergaard AF, Sun S, Veidal SS, Larsen L, Zheng Q, Suetta C, Henriksen K, Christiansen C, Karsdal MA, Leeming DJ (2013). The neo-epitope specific PRO-C3 ELISA measures true formation of type III collagen associated with liver and muscle parameters. Am J Transl Res.

[18] Sun S, Henriksen K, Karsdal MA, Byrjalsen I, Rittweger J, Armbrecht G, Belavy DL, Felsenberg D, Nedergaard AF (2015). Collagen type III and VI turnover in response to long-term immobilization. PLoS ONE.

[19] Barascuk N, Veidal SS, Larsen L, Larsen DV, Larsen MR, Wang J, Zheng Q, Xing R, Cao Y, Rasmussen LM, Karsdal MA (2010). A novel assay for extracellular matrix remodeling associated with liver fibrosis: an enzyme-linked immunosorbent assay (ELISA) for a MMP-9 proteolytically revealed neo-epitope of type III collagen. Clin Biochem.

[20] Bager CL, Gudmann N, Willumsen N, Leeming DJ, Karsdal MA, Bay-Jensen AC, Hogdall E, Balslev I, He Y (2016). Quantification of fibronectin as a method to assess ex vivo extracellular matrix remodeling. Biochem Biophys Res Commun.

[21] Chua F, Gauldie J, Laurent GJ (2005). Pulmonary fibrosis: searching for model answers. Am J Respir Cell Mol Biol.

[22] Drakopanagiotakis F, Xifteri A, Polychronopoulos V, Bouros D (2008). Apoptosis in lung injury and fibrosis. Eur Respir J.

[23] Switalla S, Lauenstein L, Prenzler F, Knothe S, Forster C, Fieguth HG, Pfennig O, Schaumann F, Martin C, Guzman CA (2010). Natural innate cytokine response to immunomodulators and adjuvants in human precision-cut lung slices. Toxicol Appl Pharmacol.

[24] Wollin L, Schuett J, Ostermann A (2015). The effect of nintedanib compared to pirfenidone on serum-stimulated proliferation of human primary lung fibroblasts at clinically relevant concentrations. Am J Respir Crit Care Med.

[25] Neuhaus V, Danov O, Konzok S, Obernolte H, Dehmel S, Braubach P, Jonigk D, Fieguth H-G, Zardo P, Warnecke G (2018). Assessment of the cytotoxic and immunomodulatory effects of substances in human precision-cut lung slices. JoVE.

[26] Mross K, Stefanic M, Gmehling D, Frost A, Baas F, Unger C, Strecker R, Henning J, Gaschler-Markefski B, Stopfer P (2010). Phase I study of the angiogenesis inhibitor BIBF 1120 in patients with advanced solid tumors. Clin Cancer Res.

[27] Eisen T, Shparyk Y, Macleod N, Jones R, Wallenstein G, Temple G, Khder Y, Dallinger C, Studeny M, Loembe AB, Bondarenko I (2013). Effect of small angiokinase inhibitor nintedanib (BIBF 1120) on QT interval in patients with previously untreated, advanced renal cell cancer in an open-label, phase II study. Invest New Drugs.

[28] Wind S, Schmid U, Freiwald M, Marzin K, Lotz R, Ebner T, Stopfer P, Dallinger C (2019). Clinical pharmacokinetics and pharmacodynamics of nintedanib. Clin Pharmacokinet.

[29] Hoyer N, Jessen H, Prior TS, Sand JMB, Leeming DJ, Karsdal MA, Attingsberg EKA, Vangsgaard GKM, Bendstrup E, Shaker SB (2021). High turnover of types III and VI collagen in progressive idiopathic pulmonary fibrosis. Respirology.

[30] Dancer RC, Wood AM, Thickett DR (2011). Metalloproteinases in idiopathic pulmonary fibrosis. Eur Respir J.

[31] Hostettler KE, Zhong J, Papakonstantinou E, Karakiulakis G, Tamm M, Seidel P, Sun Q, Mandal J, Lardinois D, Lambers C, Roth M (2014). Anti-fibrotic effects of nintedanib in lung fibroblasts derived from patients with idiopathic pulmonary fibrosis. Respir Res.

[32] Sava P, Ramanathan A, Dobronyi A, Peng X, Sun H, Ledesma-Mendoza A, Herzog EL, Gonzalez AL (2017). Human pericytes adopt myofibroblast properties in the microenvironment of the IPF lung. JCI Insight.

[33] Jenkins G, Maher TM, Cottin V, Nishioka Y, Noth I, White ES, Ittrich C, Diefenbach C, Rohr KB, Stowasser S, Selman M (2019). Effect of nintedanib on blood biomarkers in patients with IPF in the INMARK trial. Eur Respir J.

[34] Maher T, Jenkins G, Cottin V, Nishioka Y, Noth I, Selman M, Song JW, Prasse A, Ittrich C, Diefenbach C (2019). Blood biomarkers predicting disease progression in patients with IPF: data from the INMARK trial. Eur Respir J.

[35] Ronnow SR, Dabbagh RQ, Genovese F, Nanthakumar CB, Barrett VJ, Good RB, Brockbank S, Cruwys S, Jessen H, Sorensen GL (2020). Prolonged Scar-in-a-Jar: an in vitro screening tool for anti-fibrotic therapies using biomarkers of extracellular matrix synthesis. Respir Res.

[36] Lehmann M, Buhl L, Alsafadi HN, Klee S, Hermann S, Mutze K, Ota C, Lindner M, Behr J, Hilgendorff A (2018). Differential effects of nintedanib and pirfenidone on lung alveolar epithelial cell function in ex vivo murine and human lung tissue cultures of pulmonary fibrosis. Respir Res.

