# Supplementary material for: Nintedanib modulates type III collagen turnover in viable precision-cut lung slices from bleomycin-treated rats and patients with pulmonary fibrosis
Source: Respir Res. 2022 Aug 4;23:201. doi: 10.1186/s12931-022-02116-4 (PMC9351157; doi:10.1186/s12931-022-02116-4)

**Figure S1. No detectable increase in LDH in the culture supernatant of human PCLS after antifibrotic treatment**

Relative LDH values in culture supernatants from PCLS incubated with nintedanib and pirfenidone at 48 hours. Bar graphs depict mean values of LDH release summarised from all samples of nine donors ±SD. LDH measurements of one donor failed for technical reasons.

LDH, lactate dehydrogenase; PCLS, precision-cut lung slices; SD, standard deviation.


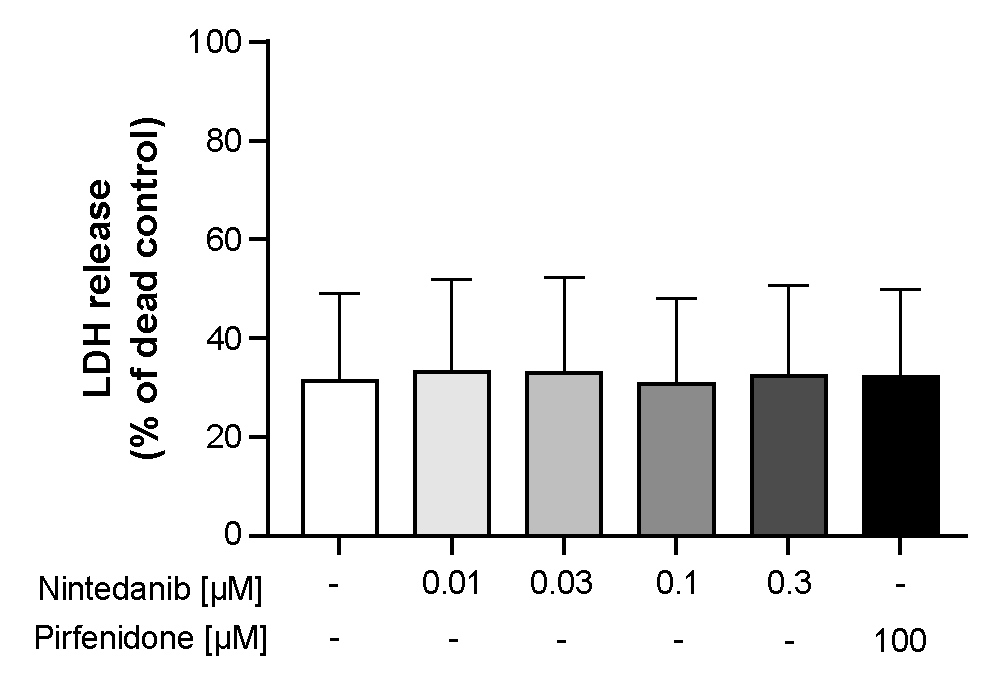

Supplement: Supplementary file 1 — Additional file 1: Figure S1. No detectable increase in LDH in the culture supernatant of human PCLS after antifibrotic treatment. [file 12931_2022_2116_MOESM1_ESM.docx]
